# Coumarin-Induced Delay of Rice Seed Germination Is Mediated by Suppression of Abscisic Acid Catabolism and Reactive Oxygen Species Production

**DOI:** 10.3389/fpls.2019.00828

**Published:** 2019-06-27

**Authors:** Bing-Xian Chen, Yuan-Xuan Peng, Jia-Dong Gao, Qi Zhang, Qin-Jian Liu, Hua Fu, Jun Liu

**Affiliations:** ^1^Argo-Biological Gene Research Center, Guangdong Academy of Agricultural Sciences, Guangzhou, China; ^2^College of Agriculture and Biology, Zhongkai University of Agriculture and Engineering, Guangzhou, China; ^3^Rice Research Institute, Guangdong Academy of Agricultural Sciences, Guangzhou, China

**Keywords:** seed germination, coumarin, abscisic acid synthesis, abscisic acid catabolism, reactive oxygen species, *Oryza sativa*, gene expression, enzyme activity

## Abstract

Abscisic acid (ABA) is a crucial phytohormone for the regulation of seed germination. The ABA content of seeds is regulated by synthesis and catabolic pathways. Coumarin, an important plant allelochemical, can inhibit seed germination effectively, although whether it is involved in the regulation of ABA content during seed germination has not been elucidated. For the study reported herein, we show that coumarin effectively inhibits rice seed germination and vivipary. We found that the ABA content gradually decreased in water-imbibed rice seeds and that the content and activity of the *Oryza sativa* 9-cis epoxycarotenoid dioxygenases (OsNCEDs), which are ABA synthases, decreased during seed germination. At the transcription level, the expression of *OsNCED1–3* appeared to decrease, whereas the expression of the ABA 8′-hydroxylase 2 and 3 genes (*OsABA8’ox2/3*) first appeared to increase and then decrease. Samples of rice seeds were also imbibed in water containing coumarin, which increased their ABA content but did not significantly increase the activity or content of their OsNCEDs or *OsNCED1–3* transcription. Interestingly, coumarin imbibition remarkably reduced *OsABA8’ox2/3* expression in rice embryos, which partially explained how coumarin increased the ABA content of germinating rice embryos. Coumarin also inhibited the accumulation of reactive oxygen species (ROS) in rice embryos and increased the activity of superoxide dismutase and catalase, which are indispensable for seed germination. These results indicate that coumarin delays seed germination by inhibiting ABA catabolism, particularly by decreasing the expression of *OsABA8’ox2/3* rather than by increasing ABA synthesis. Moreover, coumarin increases the ABA content while decreasing the ROS content in rice embryos. Our results enhance our understanding of the regulation of ABA and ROS during seed germination and provide theoretical support for application of coumarin to prevent sprouting before crop harvesting.

## Introduction

Seed germination is a critical step in the life cycle of higher plants. During germination, viable seeds develop into plants upon imbibition in water, which then allows for embryo growth until the radicle breaks through the peripheral tissue structures (endosperm and seed coat; Bewley et al., 2013).

Seed germination is regulated by various factors, including phytohormones, e.g., gibberellin, ethylene, and brassinosteroids, which promote germination, and abscisic acid (ABA), which promotes dormancy (Koornneef et al., 1998; Millar et al., 2006). For rice and *Arabidopsis*, a decrease in the ABA content of their seeds is necessary for germination (Ali-Rachedi et al., 2004; Zhu et al., 2009). In addition, many studies have shown that certain seed germination inhibitors regulate germination by altering the content of ABA in seeds. For example, glucose and copper inhibit rice seed germination by increasing the amount of ABA (Zhu et al., 2009; Ye et al., 2014). Furthermore, fluridone, a synthetic inhibitor of ABA, effectively reduces the ABA content of seeds, which promotes seed germination. Therefore, it is apparent that the ABA content of seeds determines whether germination will occur.

Accumulation of endogenous ABA in seeds depends on a dynamic balance between ABA synthesis and catabolism. In plants, ABA is synthesized directly from carotenoids, with the committed step being catalyzed by 9-cis epoxycarotenoid dioxygenase (NCED), which cleaves 9-cis xanthophyll to xanthoxin (Nambara and Marion-Poll, 2005; Rodríguez-Gacio et al., 2009). In water-stressed beans, the transcription of the key NCED gene, *PvNCED1*, and subsequent translation of its mRNA correlate directly with changes in the endogenous ABA content (Qin and Zeevaart, 1999). For *Arabidopsis* seeds, NCED mutants germinate more rapidly than do wild-type seeds (Frey et al., 2012), and ectopic expression of the sorghum NCED gene into *Arabidopsis* caused 9- to 73-fold increases in ABA levels and allowed the seed to remain deeply dormant for over 3 months (Nonogaki et al., 2014). The cytochrome P450, CYP707A2, is an ABA 8′-hydroxylase (ABA8’ox) that inactivates ABA by chemically modifying it and thereby reduces the ABA content in seeds and, in turn, relieves seed dormancy and promoting germination. Expression of *Arabidopsis thaliana* CYP707A2 (*AtCYP707A2*) in germinating *Arabidopsis* seeds is associated with a rapid decline in ABA content (Kushiro et al., 2004). In the seeds of a cyp707a2 mutant, its ABA failed to decrease in response to nitrate, which can release seed dormancy in *Arabidopsis* by reducing ABA levels, and the seeds did not germinate (Matakiadis et al., 2009).

The allelochemical coumarin, an unsaturated lactone, inhibits germination or reduces seedling growth of lettuce (Bewley et al., 2013). It induced dormancy in lettuce seeds by antagonizing gibberellin function, as the addition of gibberellin was unable to reverse coumarin-induced inhibition (Khan and Tolbert, 1966; Berrie et al., 1968). Although coumarin regulates germination, whether it acts by regulating the synthesis and/or catabolism of ABA is unclear. Coumarin inhibits catalase, superoxide dismutase (SOD), and ascorbate peroxidase (APX), all of which destroyed reactive oxygen species (ROS) in *Sorghum sudanense* seeds and thereby inhibited their germination (Wang et al., 2017) because ROS are needed to loosen the cell wall of seeds necessary for germination (Müller et al., 2009). ABA reduced ROS production during germination of imbibed rice seeds (Ye et al., 2012), but the relationship among coumarin, ABA synthesis/catabolism, and ROS production with respect to inhibition of germination is unclear.

Although the physiological and molecular mechanisms of seed dormancy and germination of *A. thaliana* seeds have been well studied, there are many unresolved issues concerning cultivation of crops in the field. How to effectively solve such issues, e.g., slow germination rates and pre-harvest sprouting (PHS), is crucial to increasing agricultural yields (Bewley et al., 2013; Nonogaki et al., 2018). We previously found that coumarin can effectively prevent rice PHS, but the physiological and molecular mechanisms involved are still unclear. For the study reported herein, we comprehensively examined the effects of coumarin on germination, ABA synthesis and its degradation, ROS production, and expression of related enzyme genes in rice seeds. We found that coumarin inhibited degradation of ABA by inhibiting OsABA8’ox activity, rather than ABA synthesis. Our results help clarify the relationship between coumarin, ABA, and ROS during seed germination. Our results also suggest that coumarin could be used to inhibit or prevent PHS in crops in the field.

## Materials and Methods

### Non-plant Materials

Coumarin, fluridone, diniconazole, ABA, nitroblue tetrazolium (NBT), 3,3′-diaminobenzidine hydrochloride (DAB), 3,3′,5,5′-tetramethylbenzidine (TMB), and 2,3,5-triphenyl tetrazolium chloride (TTC) were purchased from Sigma-Aldrich (USA). Water was doubly distilled before use.

### Plant Materials, Seed Germination, and Rice Pre-harvest Sprouting Tests

Rice seeds (*O. sativa* ssp*. indica* cv. R998) with their glume intact were placed into transparent plastic germination boxes (12 cm × 12 cm × 6 cm) containing two layers of filter paper soaked in water and with one of the treatments shown in Supplementary Figure S1. The final volume of each sample was 25 ml. The seeds were incubated in a growth chamber at 28 ± 1°C under a 16-h light/8-h dark photocycle. The light intensity was 190 μmol/m^2^/s by photosynthetic photon flux density. Seeds were counted at 3-h intervals between 36 and 48 h.

To test for rice vivipary, seeds were soaked in water containing 1, 5, 10, or 20 mM coumarin for 1 min, then removed and allowed to dry at room temperature before being imbibed in water to determine the percentage of seeds that would germinate. And then a suitable concentration of aqueous coumarin that was found to effectively inhibit germination, i.e., 10 mM coumarin, was sprayed onto mature seeds on rice plants (15 days before harvest) that had been growing in a greenhouse. The control group (mature seeds on rice plants) was sprayed with water. The temperature and humidity in the greenhouse were kept at 38 ± 2°C and 95 ± 2%. The treated rice plants were harvested 15 days after being sprayed.

Seeds with protruding radicles were regarded as having completed germination. The number of germinated seeds at each time point was converted to a percentage, and the mean value ± SE of three biological replicates of 100 seeds each was calculated. Seeds were photographed using a stereomicroscope (SteREO Lumar V12, Zeiss, Germany).

### Measurement of Endogenous Abscisic Acid Levels

Rice seed embryos (1 g) were ground into a powder in liquid nitrogen, and 10 ml of isopropanol/hydrochloric acid (3:1) containing 8 μl of 50 ng/ml ABA as an internal standard was added into the powder, and the mixture was then shaken at 4°C for 30 min. Next, 20 ml of dichloromethane was added into the mixture, which was then shaken at 4°C for 30 min. The mixture was centrifuged at 11,255 × *g* for 5 min at 4°C, after which the upper portion of the supernatant was discarded and the lower organic phase retained. The organic phase was dried under nitrogen in the dark and then dissolved in 400 μl of methanol containing 0.1% formic acid. The solution was passed through a 0.22-μm filter, and the ABA content was determined with HPLC (Model 1,290, Agilent, USA) coupled with tandem mass spectrometry (MS/MS, SCIEX-6500Qtrap, AB SCIEX, USA). The mean value ± SE of three biological replicates is reported.

### 9-Cis Epoxycarotenoid Dioxygenase Content and Activity Assays

Phosphate-buffered saline (pH 7.4, 9 ml) was added into 1 g of embryos, which were then homogenized by grinding with a mortar and pestle. The homogenate was centrifuged at 600 × *g* and 4°C for 5 min, after which the supernatant was retained. The sample was then subjected to ELISA using reagents from a Plant NCED ELISA kit according to the manufacturer’s instructions (Beijing Chenglin Biotechnology Co., Ltd., Beijing, China). The *A*_450_ value of the labeled antibody-antigen complex was measured using a full-wavelength ELASA instrument (Varioskan Flash, Thermo, USA) to determine the concentration and activity of NCED. The mean value ± SE of three biological replicates was calculated.

### Phylogenetic Relationships and Structural Domain Analysis

To better understand the evolution of the NCED and ABA8’ox gene families in monocot and eudicot, we generated unrooted maximum-likelihood phylogenetic trees. We selected ABA metabolism-related genes from rice Nipponbare including *OsNCED1* (AY838897), *OsNCED2* (AY838898), *OsNCED3* (AY838899), *OsNCED4* (AY838900), *OsNCED5* (AY838901), *OsABA8’ox1* (i.e., *OsCYP707A5*, AB277270), *OsABA8’ox2* (i.e., *OsCYP707A6*, NM_001068556), and *OsABA8’ox3* (i.e., *OsCYP707A7*, NM_001069901) according to the previous studies (Saika et al., 2007; Zhu et al., 2009; Liu et al., 2011). In addition, NCED and ABA8’ox genes of other plant species are also included according to published reports (Supplementary Table S1). The sequences of *O. sativa, A. thaliana,* and other plant species were acquired from the Rice Annotation Project Database, the *Arabidopsis* Information Resource (TAIR v10.02), or NCBI. Alignment of sequences and phylogenetic analysis were carried out by using Clustal W with default parameters and MEGA7 with maximum-likelihood method. Finally, the phylogenetic tree was perfected by ITOL^1^. The Pfam and SMART databases^2^ were used to analyze the functional domains of the identified NCED and ABA8’ox candidates described by Chang et al. (2016).

### *In silico* Expression Profiles (Heat Maps) and Quantitative Polymerase Chain Reaction of 9-Cis Epoxycarotenoid Dioxygenase and ABA8’ox mRNAs

We used the Os_51k microarray data in the Genevestigator V3 database to analyze the expression profiles of rice NCED and ABA8’ox genes by constructing heat maps from the datasets (Hruz et al., 2008).

To characterize the expression profiles of the OsNCED and OsABA8’ox genes by quantitative polymerase chain reaction (qPCR), 30 embryos from seeds that had been incubated in water or 1 mM coumarin for six imbibition times (6, 12, 18, 24, 36, or 48 h) were extracted and immediately frozen at −80°C. Total RNA was isolated using Column Plant RNAout 2.0 kit reagents (TIANDZ, China), and qPCR was performed as described (Chen et al., 2016). The gene-specific primers (Supplementary Table S2) were designed to avoid conserved regions, introns, and the single exon-exon junction. OsGAPDH1 mRNA (RAP-DB ID: Os02g0601300) expression served as the internal control. The mean value ± SE for each experiment of three biological replicates was calculated.

### Histochemical Localization of O_2_^−^ and H_2_O_2_

We used NBT and DAB, respectively, to stain seeds for O_2_^−^ and H_2_O_2_ as described (Li et al., 2017). After rice seeds had imbibed water or 1 mM coumarin for 6, 18, or 36 h, five whole seeds and five half-granule seeds each containing an embryo were removed and incubated with 1 mM NBT in 10 mM Tris-HCl (pH 7.0) or 1 mg/ml DAB (pH 3.8) at room temperature for 30 min, then washed with double-distilled water, and photographed under a SteREO Lumar V12 stereomicroscope.

### TTC Staining

TTC (0.5 g) was dissolved in 2 ml of ethanol, which was then added into 100 ml of double-distilled water, and stored at 4°C in the dark until used. After incubation in water or 1 mM coumarin for 6, 18, or 36 h, five whole seeds and five half-granule seeds each containing an embryo were stained with 0.5% TTC at 35°C for 3 h, then washed three times with water, and photographed as described above.

### Histochemical Detection of Peroxidase Activity

Peroxidase is the key enzyme for production of •OH, and the level of peroxidase activity indirectly reflects the production and accumulation of •OH. We detected peroxidase activity histochemically by TMB staining as described (Li et al., 2017). Briefly, after rice seeds had been imbibed in water or 1 mM coumarin for 6, 18, or 36 h, five whole seeds and five half-granule seeds (each containing an embryo) were incubated in 0.2% TMB, 1 mM H_2_O_2_, 20 mM potassium phosphate (pH 6.5) at room temperature for 30 min, then washed in water and photographed as described above.

### Superoxide Dismutase, Catalase, and Ascorbate Peroxidase Activity Measurements

A crude extract of the enzymes from rice seed embryos was prepared according to Ye et al. (2014). Frozen seeds (0.5 g) were homogenized on ice with 1 ml of 50 mM potassium phosphate (pH 7.0), 1 mM EDTA, and 1% polyvinylpyrrolidone. Each homogenate was centrifuged at 12,000 × *g* for 30 min at 4°C, and each supernatant was retained for enzyme assays. Protein content was determined according to the method of Bradford (1976) with bovine serum albumin as the standard. The activities of SOD, catalase, and APX were determined spectrophotometrically according to Ye et al. (2014). The mean value ± SE for each experiment of three biological replicates was calculated.

### Statistical Analysis

Data are presented as the mean ± SE of three replicates. One-way analysis of variance was used to compare mean values, and when significant, differences between individual means were compared with the Fisher’s least-significant difference test. Student’s *t* test was conducted to evaluate variances in the changes in germination percentage, ABA content, and expression levels of *OsNCED1–3* and *OsABA2/3*.

## Results

### Germination of Rice Seeds Is Inhibited by Coumarin and by Inhibitors of Abscisic Acid Catabolism but not by Inhibitors of Abscisic Acid Synthesis

**Figure 1:**
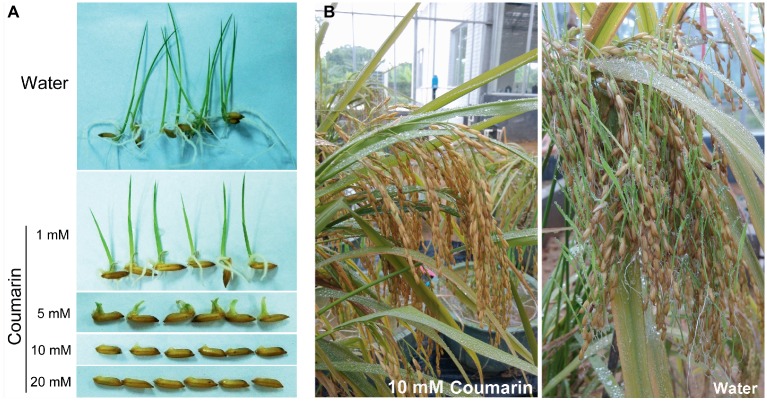
Morphologies of germinating seeds and pre-harvest-sprouted rice. **(A)** Morphologies of rice seeds imbibed in water or in 1, 5, 10, or 20 mM coumarin. **(B)** The extent of pre-harvest-sprouted rice sprayed with 10 mM coumarin (left) or water (right) under greenhouse conditions of 38 ± 2°C and 95 ± 2% humidity.

A relatively small dose of coumarin can inhibit the germination of lettuce (Berrie et al., 1968), soybean (Colpas et al., 2003), and *Sorghum sudanense* seeds (Wang et al., 2017). We soaked rice seeds in 1, 5, 10, or 20 mM coumarin for 1 min and then transferred them into water for germination. Only the seeds treated with 1 or 5 mM coumarin germinated, but their progression to the seedling stage was inhibited compared with control seeds. Conversely, seeds treated with 10 or 20 mM coumarin were unable to germinate (Figure 1A). In the greenhouse, we mimicked the high-temperature and high-humidity conditions for field-grown rice at the pre-harvest stage. When we sprayed the rice with 10 mM coumarin, their vivipary was inhibited (2.8% vs. 35.4% for the water-sprayed controls; Figure 1B).

**Figure 2:**
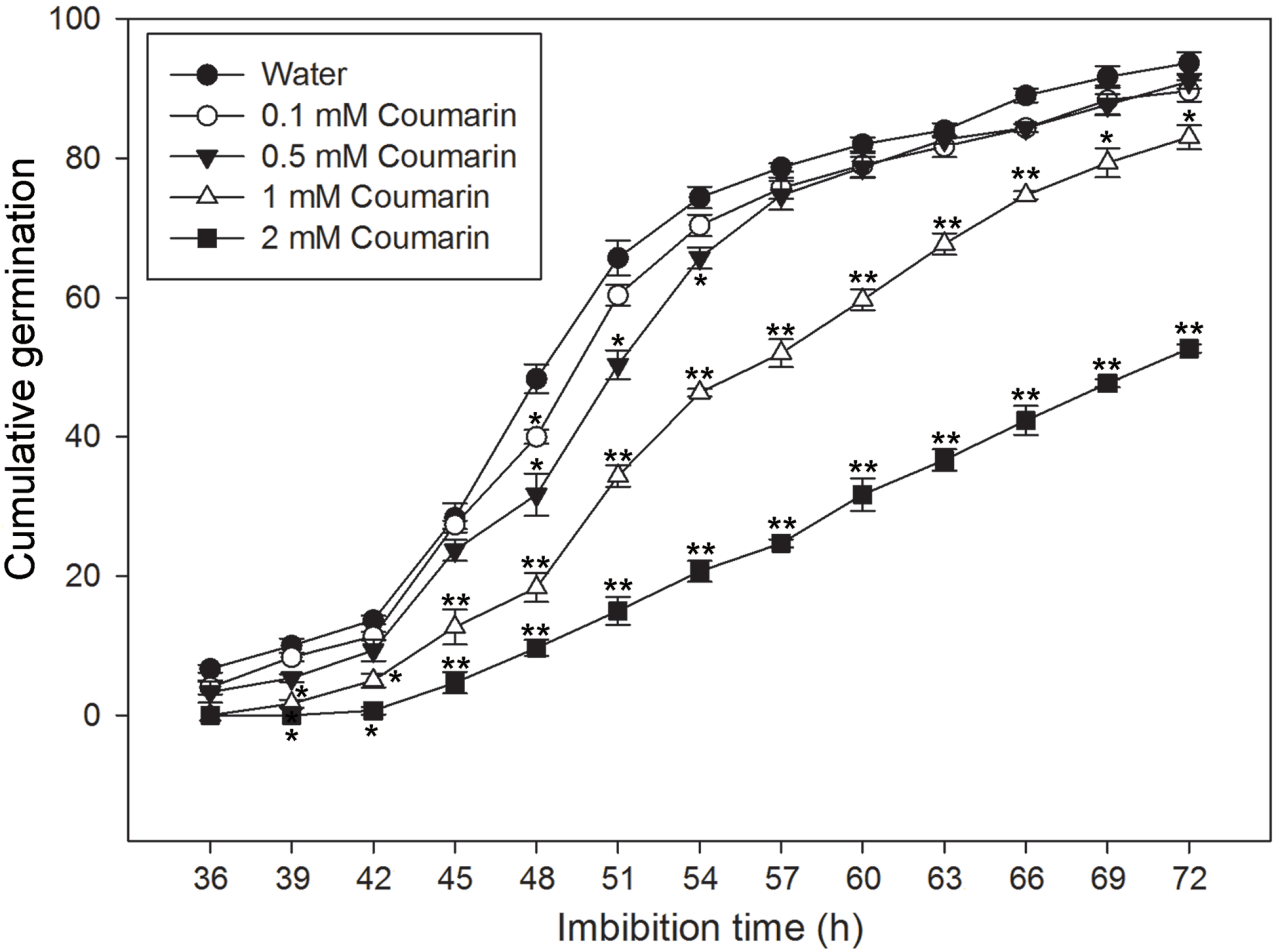
Germination time courses for rice seeds imbibed in water, or in 0.1, 0.5, 1, or 2 mM coumarin. The number of seeds that had germinated was determined every 3 h for 72 h, and the results are presented as the cumulative germination percentage. Data represent the mean ± SE of three biological replicates of 100 seeds each. Significant differences in the data of germination percentage in 0.1, 0.5, 1, and 2 mM coumarin from those in water at each imbibition time point were assessed by Student’s *t* test (^*^p < 0.05; ^**^p < 0.01).

To further investigate the effect(s) of different concentrations of coumarin on rice seed germination, we carried out a germination time-course experiment, which revealed that rice seeds began to germinate at 36 h when imbibed in water. Conversely, exposure to coumarin inhibited rice seed germination, and this inhibition was more obvious as the coumarin concentration increased. Notably, 1 or 2 mM coumarin decreased the number of seeds that germinated throughout the experiment, i.e., at each time point, and delayed the initial germination time by 3 and 9 h, respectively. After 72 h, the germination percentages for seeds exposed to 1 or 2 mM coumarin were 83 and 53%, respectively, of the control seeds (Figure 2).

Fluridone is a potent inhibitor of ABA synthesis (Nambara and Marion-Poll, 2005; Martínez-Andújar et al., 2011), and diniconazole is a potent inhibitor of ABA catabolism (Kitahata et al., 2005). Fluridone (50 μM) increased rice seed germination by ~8% at 48 h and by ~9% at 60 h. Diniconazole (10 μM) decreased rice seed germination by 12% at 48 h and 9% at 60 h (Supplementary Figure S1). When rice seeds were imbibed in 0.1, 0.5, 1, or 2 mM coumarin plus 50 μM fluridone, seed germination was not significantly altered compared with seeds imbibed only in coumarin. Conversely, germination was further inhibited when the seeds were imbibed in 2 mM coumarin plus 10 μM diniconazole (5% germination) compared with coumarin alone (10% germination; Supplementary Figure S1). These results suggested that coumarin-mediated inhibition of germination and fluridone-mediated stimulation of germination may not offset each other, whereas coumarin and diniconazole may act synergistically to inhibit germination.

### Coumarin Significantly Increases the Abscisic Acid Content of Rice Embryos but Does not Substantially Affect 9-Cis Epoxycarotenoid Dioxygenase Content or Activity

**Figure 3:**
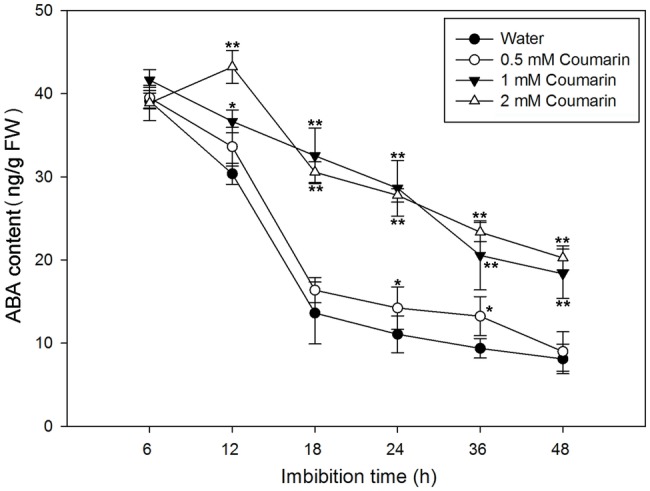
Changes in ABA levels in imbibed seeds in the absence or presence of coumarin. Seeds were imbibed at 28°C in water, or in 0.5, 1, or 2 mM coumarin. Seed samples were collected at different time points and stored at −80°C for ABA content determination. ABA was quantified with HPLC. Data represent the mean ± SE of three biological replicates each of 1 g of embryos. Significant differences in the data of ABA content in 0.5, 1, and 2 mM coumarin from those in water at each imbibition time point were assessed by Student’s *t* test (^*^p < 0.05; ^**^p < 0.01). FW, fresh weight.

During imbibition of water by *Arabidopsis* and barley seeds, the ABA content gradually decreases (Millar et al., 2006). To determine ABA levels in rice seeds, we first isolated embryos from germinating seeds that had imbibed water or 0.5, 1, or 2 mM coumarin for 6 to 48 h. After which, the ABA concentrations in the embryos were measured. For the water-imbibed seeds, the ABA content decreased rapidly between 6 and 18 h and slowed thereafter (Figure 3). The ABA content decreased slightly in seeds imbibed in 0.5 mM coumarin, and these seeds had an ABA-content time course similar to that of the water-imbibed seeds. Conversely, imbibition of 1 or 2 mM coumarin substantially enhanced the relative ABA content of the seeds at all time points in comparison with that of the water-imbibed seeds, although the ABA content of the seeds that had imbibed 1 or 2 mM coumarin also gradually and continuously decreased except for the 12-h time point of seeds imbibed with 2 mM coumarin (Figure 3).

**Figure 4:**
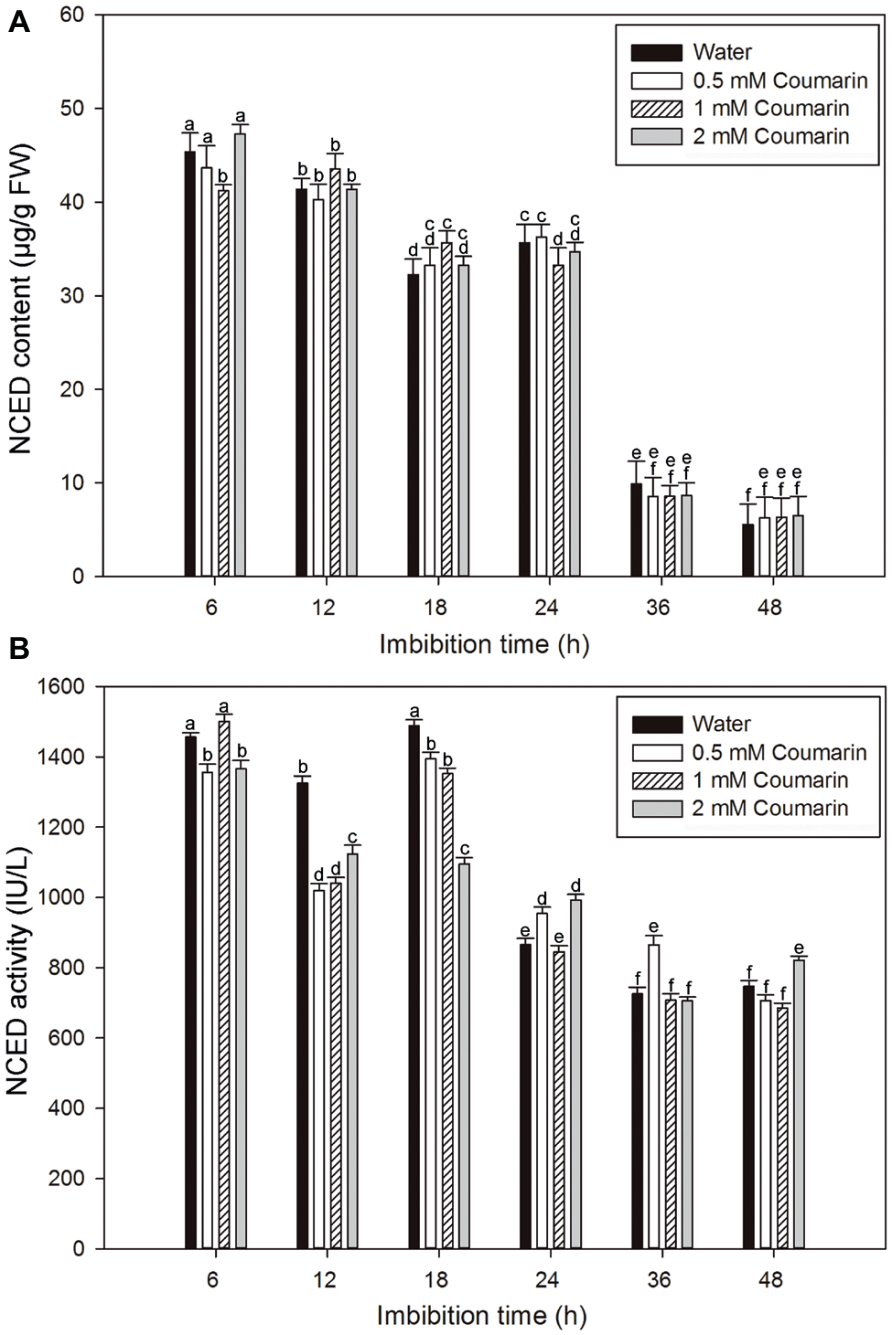
Changes in NCED content and activity in imbibed seeds in the absence or presence of coumarin. Seeds were imbibed at 28°C in water, or in 0.5, 1, or 2 mM coumarin. Seed samples were collected at different times and stored at −80°C for NCED content **(A)** and NCED activity **(B)** determination. NCED content and activity were determined ELISA. Data represent the mean ± SE of three biological replicates each of 1 g of embryos. Means denoted by the same letter did not significantly differ at *p* < 0.05 according to Fisher’s least significant difference test.

NCED is the key enzyme in ABA synthesis, and changes in NCED activity are closely related to the ABA content of plant seeds (Yamaguchi et al., 2007). An ELISA study was performed to explore changes in the NCED level during the production of ABA in rice embryos. For embryos of rice seeds imbibed in water, both the NCED content and activity remained at relatively high levels from 6 to 24 or 18 h and then remarkably decreased afterward, and similar results were obtained for seeds imbibed in coumarin (Figures 4A,B). However, 0.5, 1, or 2 mM coumarin did not substantially promote or inhibit the NCED content during the whole imbibition process (Figures 4A,B).

### Identification and Phylogenetic Analysis of the Rice 9-Cis Epoxycarotenoid Dioxygenase and ABA8’ox Genes

**Figure 5:**
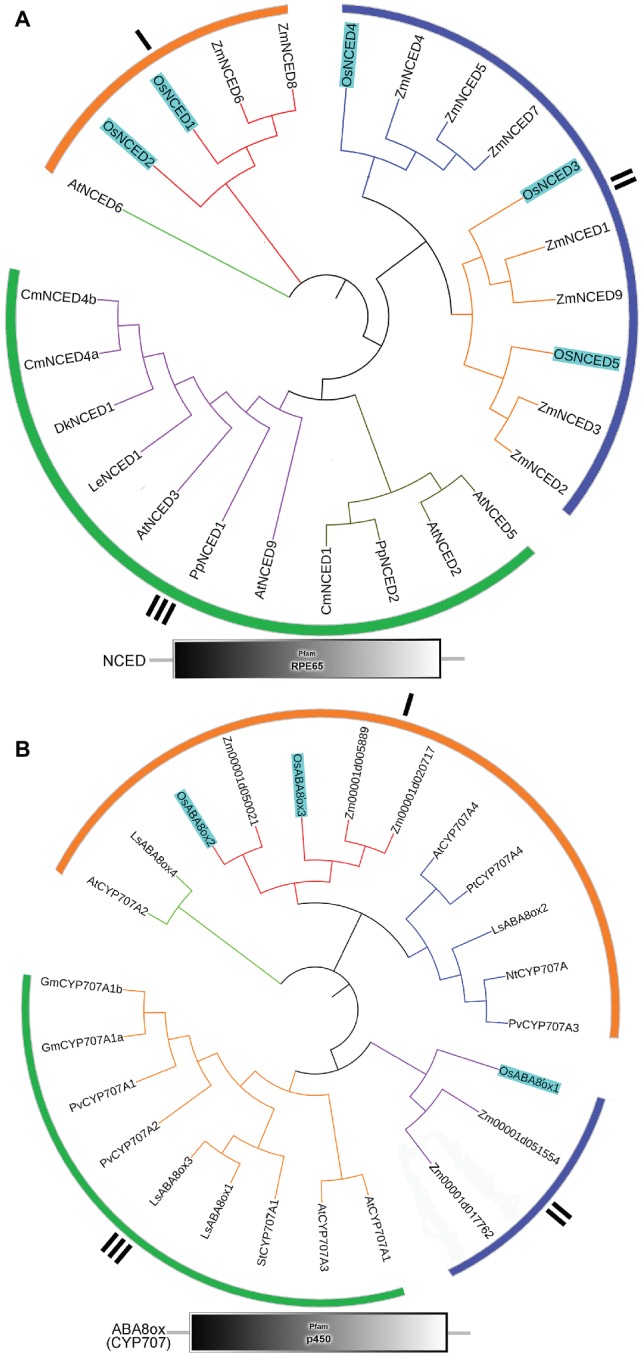
Phylogenetic trees and predicted domains for the NCED and ABA8’ox families. **(A)** An unrooted maximum-likelihood phylogenetic tree based on an alignment of the amino oxidase sequences of NCEDs from the following plant species: *O. sativa, A. thaliana, Zea mays, Solanum lycopersicum, Prunus persica, Diospyros kaki, Cucumis melo*, and *Chrysanthemum × morifolium*. The sequences used to build the tree and their GenBank accession numbers are displayed in Supplementary Table S1. Domain organization of the NCEDs from the aforementioned plants is shown below the tree. **(B)** An unrooted maximum-likelihood phylogenetic tree based on the alignment of the amino oxidase sequences of OsABA8’oxs from the following plant species: *O. sativa, A. thaliana, Zea mays, Populus trichocarpa, Lactuca sativa, Glycine max, Nicotiana tabacum, Solanum tuberosum*, and *Phaseolus vulgaris*. The sequences used to build the tree and their GenBank accession numbers are displayed in Supplementary Table S1. Domain organization of ABA8’oxs in the abovementioned plants is shown below the tree.

To identify the NCED and ABA8’ox genes present in rice, we used the *Arabidopsis* NCED and CYP707A sequences as queries for searches of the rice genome database. For multiple-sequence alignment, we also included NCED and ABA8’ox sequences from seven other plant species (Figure 5). All NCED and ABA8’ox sequences included all their domains, e.g., an RPE65 domain and a p450 domain, for the NCED and ABA8’ox sequences, respectively (Figure 5). To investigate the evolutionary relationships among these proteins, we generated two unrooted phylogenetic trees one containing the NCED sequences and the other containing the ABA8’ox sequences from the aforementioned species. According to the topological structures of the trees, the NCED and the ABA8’ox families of monocotyledon and dicotyledon plants can each be divided into three subfamilies, denoted Sub I, II, and III. For both enzymes, those in the Sub I family are found in monocotyledonous and dicotyledonous plants, whereas those in Sub II and III families belong to monocotyledons or dicotyledons, respectively (Figure 5). The OsNCED1/2 sequences are in Sub I, whereas the OsNCED3*–*5 sequences are in the monocotyledon-specific Sub II family (Figure 5A). The OsABA8’ox2/3 sequences belong to the Sub I family, whereas the OsABA8’ox1 sequence is in the monocotyledon-specific Sub II family (Figure 5B). These results suggested that, among monocotyledonous and dicotyledonous plants, each of the NCED and ABA8’ox families has evolved from a single (but different) ancestor. During the evolution of monocotyledons and dicotyledons, these gene families diverged with genetic duplication to generate their own distinct subfamilies.

### The Expression Profiles of OsNCED and OsABA8’ox Genes Differed Substantially During Rice Germination, and the Transcript Levels of *OsABA8’oxs* in Rice Embryos Decrease After Imbibition With Coumarin

We examined the expression patterns of *OsNCED1–5* and *OsABA8’ox1–3* by displaying the rice microarray data in the Genevestigator database as heat maps. We found substantial differences in their expression during different development stages of rice (Supplementary Figure S2). In seedlings, *OsNCED3* and *OsABA8’ox3* were each expressed at a higher level than those of the other genes, suggesting that these two genes may be involved in seed germination and establishment of seedlings (Supplementary Figure S2A). In comparison with the individual expression the OsNCED genes, except for *OsNCED3*, expression of *OsABA8’ox2/3* was greater, and the expression of *OsABA8’ox2/3* decreased gradually as germination progressed (Supplementary Figure S2B).

**Figure 6:**
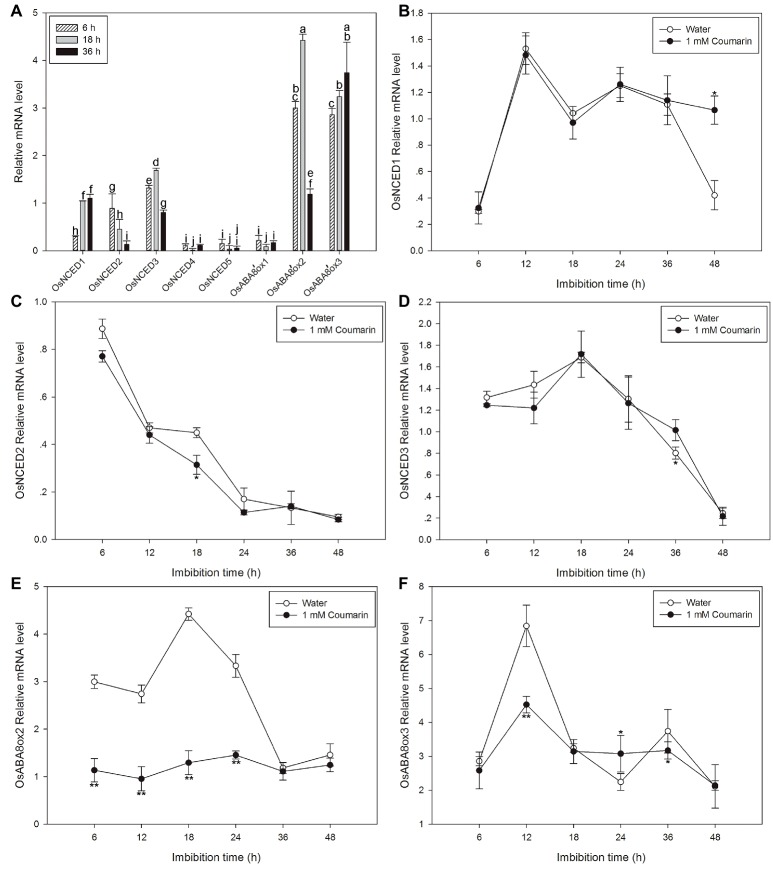
Changes in the expression profiles for OsNCED and OsABA8’ox genes during rice seed germination as assessed with qPCR. **(A)** The expression levels of *OsNCED1–5* and *OsABA8’ox1–3* in embryos assayed by qPCR after their seeds had been imbibed in water for 6, 18, or 36 h. Data represent the mean ± SE of three biological replicates, each with 30 embryos (0.1 g per replicate). Expression patterns for *OsNCED1* **(B)**, *OsNCED2* **(C)**, *OsNCED3* **(D)**, *OsABA8’ox2* **(E)**, and *OsABA8’ox3* **(F)** in embryos as assayed by qPCR after seed imbibition of water or 1 mM coumarin at 6, 12, 18, 24, 36, and 48 h. Data represent the mean ± SE of three biological replicates, each with 30 embryos (0.1 g per replicate). Means denoted by the same letter did not significantly differ at *p* < 0.05 according to Fisher’s least significant difference test **(A)**. Significant differences in the data of relative mRNA level in 1 mM coumarin from those in water at each imbibition time point were assessed by Student’s *t* test (**B–F**, ^*^p < 0.05; ^**^p < 0.01).

Next, qPCR was used to further characterize the expression profiles of *OsNCED1–5* and *OsABA8’ox1–3*. The results were partially consistent with the expression patterns that were based on the microarray data. The discrepancies may be a consequence of different rice varieties and/or materials. *OsNCED1–3* were expressed at higher levels than *OsNCED4–5*, and *OsABA8’ox2/3* were expressed at higher levels than *OsABA8’ox1* (Figure 6A). Therefore, the expression profiles of *OsNCED1–3* and *OsABA8’ox2/3* were then examined throughout the course of germination (at 6, 12, 18, 24, 36, and 48 h) by qPCR when seed was imbibed in water and 1 mM coumarin. The expression of *OsNCED1/3* initially increased, peaked between 12 and 18 h, and then decreased gradually (Figures 6B,D). The expression of *OsNCED2* began to gradually decrease at 6 h and reached its lowest value at 48 h of imbibition (Figure 6C). Notably, 1 mM coumarin had little consistent effect on the expression of *OsNCED1–3*, as it inhibited or enhanced their expression at specific time points. The expression patterns of *OsABA8’ox2/3* were similar, showing first an increase and then a decrease, with a peak value at 18 h for *OsABA8’ox2* and at 12 h for *OsABA8’ox3*. Interestingly, 1 mM coumarin strongly inhibited the transcription of both genes, particularly that of *OsABA8’ox2* between 6 and 24 h (2.3–3.4-fold) and that of *OsABA8’ox3* at 12 h (1.5-fold; Figures 6E,F).

### Coumarin Has a Negligible Effect on Seed Viability but Suppresses Accumulation of Endogenous Reactive Oxygen Species and Enhances the Activities of Reactive Oxygen Species-Degrading Enzymes

**Figure 7:**
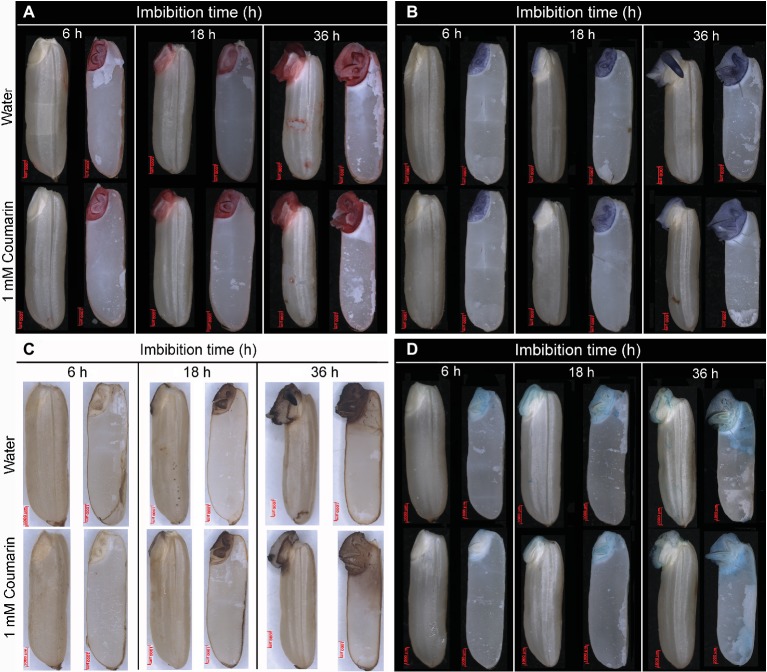
Histochemical staining to assess seed *via*bility, O_2_^−^ and H_2_O_2_ content, and peroxidase activity during germination of rice seeds in water or coumarin. Seeds were imbibed in water or 1 mM coumarin for 6, 18, or 36 h, after which whole seeds and slit half seeds were stained. **(A)** Seed viability was assessed by staining with TTC. **(B)** O_2_^−^ content was assessed by staining with NBT. **(C)** H_2_O_2_ content was assessed by staining with DAB. **(D)** Peroxidase activity was assessed by staining with TMB.

TTC staining is an effective method for determining seed viability. Seeds that are hardy with respect to viability also tend to undergo germination (Batty et al., 2001). When lettuce seeds are chemically poisoned by sodium dichloroisocyanurate, their viability declines and their germination potential is expected to be inhibited (Zhang et al., 2014). To investigate the effect of coumarin on seed viability, we stained seeds imbibed in water or coumarin with a TTC solution and found that the coumarin-treated seeds were stained to a similar extent as those imbibed in water (Figure 7A), suggesting that although coumarin inhibits seed germination, its mechanism does not involve reducing seed viability.

ROS are critical for seed germination. We used NBT and DAB staining to detect O_2_^−^ and H_2_O_2_ accumulation during rice seed germination. The activity of peroxidase, which can catalyze the conversion of O_2_^−^ and H_2_O_2_ to •OH in seeds, was detected by TMB staining (Müller et al., 2007), thereby indirectly reflecting the accumulation of •OH (Zhang et al., 2014). When rice seeds were imbibed in water, the accumulation of O_2_^−^ and H_2_O_2_ in their embryos gradually increased during germination, especially by 36 h when the first seed had completed germination; by comparison, the embryos from seeds imbibed in coumarin were much less intensely stained (Figures 7B,C), indicating that coumarin had an inhibitory effect on the production of O_2_^−^ and H_2_O_2_. Conversely, no substantial change in peroxide activity was found for the embryos from the water- and coumarin-imbibed seeds during the germination process, indicating that their •OH content was not substantially affected by the imbibition process and coumarin (Figure 7D).

**Figure 8:**
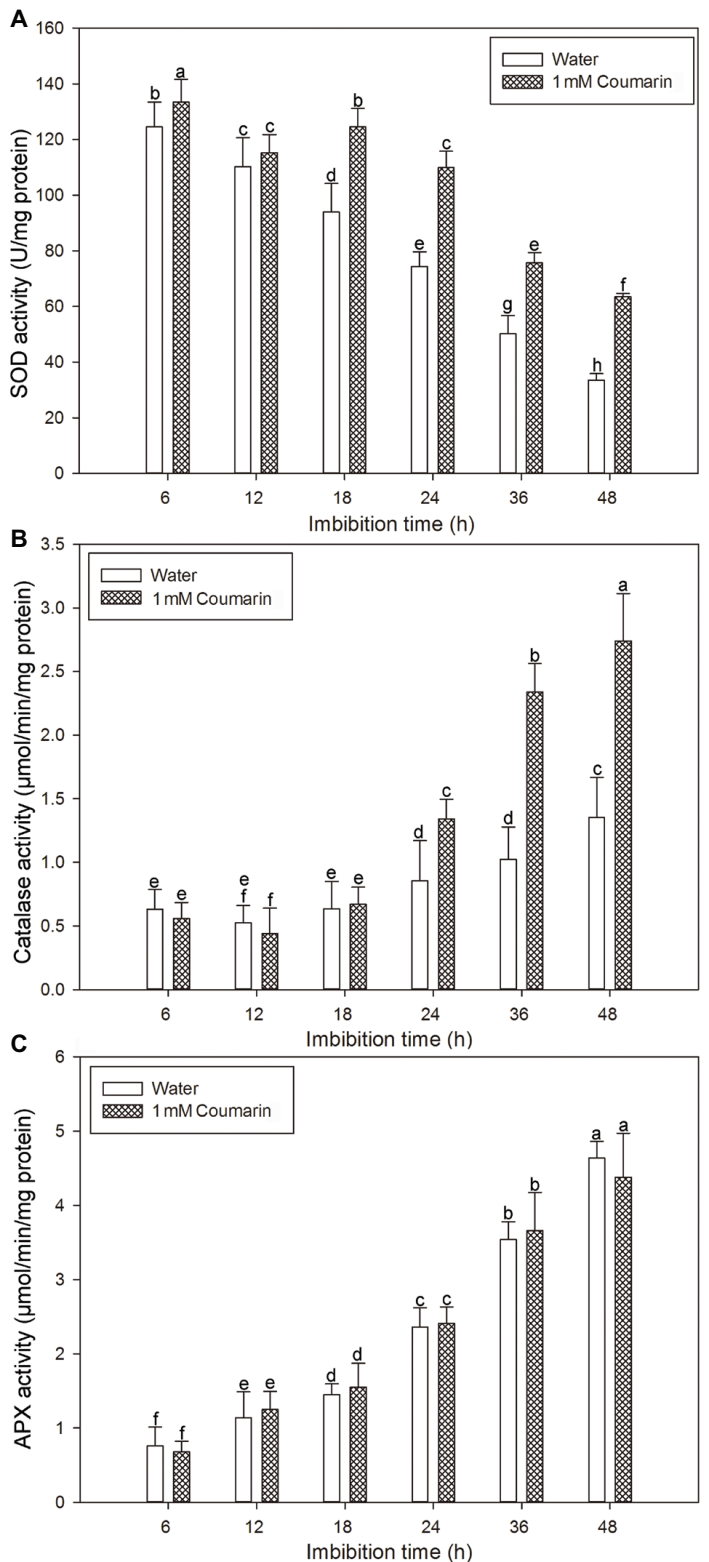
Changes in the activities of ROS-degrading enzymes in rice seeds. Effects of coumarin on the activity of SOD **(A)**, catalase **(B)**, and APX **(C)** in embryos assayed *via* spectrophotometry after their seeds had been imbibed in water or 1 mM coumarin for 6, 12, 18, 24, 36, or 48 h. Data represent the mean ± SE of three biological replicates, each with 150 embryos (0.5 g per replicate). Means denoted by the same letter did not significantly differ at *p* < 0.05 according to Fisher’s least significant difference test.

Many antioxidant enzymes, e.g., SOD, catalase, and APX play key roles in scavenging ROS in plants (Ye et al., 2014). The results shown in Figure 7 indicated that coumarin inhibited the accumulation of ROS in seeds, but what is its effect(s) on ROS-degrading enzymes? To answer this question, we examined the activities of ROS-scavenging enzymes, i.e., catalase, SOD, and APX, in imbibing seeds. The activities of catalase and APX gradually increased and peaked at 48 h, whereas the activity of SOD gradually decreased during germination of seeds imbibed in water. For seeds imbibed in the presence of coumarin, the SOD and catalase activities increased, especially between 24 and 48 h of germination, but the APX activity in those seeds was essentially unchanged compared with that of water-imbibed seeds (Figure 8). The enhanced SOD and catalase activities found for embryos from coumarin-imbibed seeds correlated with the inhibition of ROS accumulation found for those embryos.

## Discussion

### Both Abscisic Acid Synthesis and Catabolism Play Essential Role for Seeds Germinating in Water, Whereas Abscisic Acid Catabolism May Be More Important for Seeds Germinating in Coumarin

The ABA content in seeds is regulated by the relative rates of its synthesis and degradation. In corn seeds, when phytoene dehydrogenase or NCED activity in the synthesis pathway is inhibited, the seed will sprout prior to harvest (Robichaud et al., 1980; Durantini et al., 2008). *Arabidopsis* seeds from *cyp707a2*, a CYP707A2-type mutant that degrades ABA slowly, showed a delayed germination phenotype (Kushiro et al., 2004; Millar et al., 2006). We found that fluridone (an inhibitor of ABA synthesis) and diniconazole (an inhibitor of ABA degradation) promoted and inhibited germination, respectively, to a similar extent (Supplementary Figure S1). In combination with the result of changes in ABA content of seeds that gradually decreased at the beginning of the imbibition (Figure 3), it seems that a decrease in the ABA content of the seeds was a prerequisite for germination. These findings are similar to those found for maize and *Arabidopsis* (Millar et al., 2006; Durantini et al., 2008). For the expression profiles of *OsNCED1–5, OsNCED2/3* were highly expressed and gradually decreased from 18 to 48 h and that the changes in the expression of those two genes were consistent with the changes in NCED activity and ABA content. Conversely, the expression of *OsABA8’ox2/3* gradually increased during early seed imbibition, peaked at 18 and 12 h, respectively, and then decreased (Figures 7E,F). These results may explain the changes in ABA content, as substantial OsABA8’ox activity is needed initially during seed imbibition for rapid ABA degradation, which prepares seeds for germination.

The content of ABA in plants is controlled by ABA biosynthesis and catabolism, which are, in turn, affected by environmental factors such as water availability, temperature, and light exposure (Nambara and Marion-Poll, 2005). Glucose, mannitol, and copper can effectively increase the ABA content of water-imbibed rice seeds (Zhu et al., 2009; Ye et al., 2014). However, the effect of coumarin on ABA synthesis and catabolism during seed germination had seldom been investigated prior to this report. We found that coumarin had a direct dose-dependent effect on germination inhibition (Figure 2), which was consistent with our finding that the dose-dependent effect of coumarin on the ABA content of seeds was also direct (Figure 3). If coumarin inhibits germination by increasing ABA synthesis, which would increase ABA content in the absence of increased degradation, then it might be expected that NCED-type activity and expression of OsNCED genes should also be upregulated by coumarin. Interestingly, however, coumarin did not substantially increase OsNCED activity or content, or *OsNCED* transcription. Conversely, OsNCED gene expression and enzymatic activity were downregulated at certain imbibition times (Figures 4, 6). These results were confirmed by showing that the percentage of germinated rice seeds that had been imbibed in coumarin and the ABA-synthesis inhibitor, fluridone, was not increased compared with seeds imbibed in coumarin alone (Supplementary Figure S1).

Coumarin significantly inhibited the expression of *OsABA8’ox2/3* in the ABA catabolic pathway (Figures 7E,F). The low-level expression of these genes led to decreased OsABA8’ox activity and, consequently, a decreased rate of ABA degradation and increased ABA accumulation in comparison with water-imbibed seeds. These results are consistent with those showing that the ABA content was relatively increased in coumarin-imbibed seeds in comparison with those imbibed in water (Figure 3). In addition, in the presence of coumarin and diniconazole, seed germination was substantially inhibited compared with germination of seeds that were coumarin imbibed (Supplementary Figure S1). Given the above discussion, we believe that inhibition of rice seed germination may be a consequence of inhibition of ABA catabolism by coumarin. Consequently, coumarin may inhibit rice germination by upregulating expression of *OsABA8’ox2/3*, thereby increasing the ABA content. Our results are consistent with the observations that copper and glucose inhibit seed germination by suppressing ABA catabolism (Zhu et al., 2009; Ye et al., 2014).

### Coumarin May Regulate Endogenous Abscisic Acid Content and Reactive Oxygen Species Accumulation

Numerous studies have shown that an excessive accumulation of ROS is the underlying cause of oxidative stress in plants (Sharma et al., 2012). ROS also serve as signaling molecules during plant growth and in the development and responses to biotic and abiotic stresses (Fujita et al., 2006). Recently, however, ROS had been shown to directly act on plant cell-wall polysaccharides by breaking their glycosidic bonds, which cause the cell wall to relax, thereby promoting cell division and elongation (Zhang et al., 2014). During seed germination of dicotyledonous plants, such as cress and lettuce, ROS accumulate in their micropylar endosperm and radicle, led to endosperm weakening and radicle elongation, thereby promoting seed germination (Müller et al., 2009). Certain cell-wall hydrolases and non-enzymatic substances, such as ROS and expansin, are needed for these processes (González-Calle et al., 2015). We previously found that ROS accumulated in the radicle and coleorhiza of rice seeds during germination and that ROS production and the number of germinated seeds decreased in the presence of ROS inhibitors, i.e., diphenyleneiodonium chloride and guazatine (Li et al., 2017). Therefore, for this study, we also wanted to explore the effect(s) of coumarin on ROS accumulation in germinating rice seeds. For the coumarin-imbibed seeds, less ROS, as O_2_^−^ and H_2_O_2_, accumulated in embryos than in the embryos from water-imbibed seeds, although the peroxidase activity was not substantially affected, indicating that the •OH concentration was not much affected. These results indicated that O_2_^−^ and H_2_O_2_ might be the main types of ROS involved in germination. Seed viability can affect ROS accumulation in seeds to a certain extent. For example, when lettuce seeds were imbibed in a 0.3% sodium dichloroisocyanurate solution, the observed decrease in ROS accumulation may have been caused by a decrease in the micropylar endosperm and radicle viability (Zhang et al., 2014). We found that coumarin did not reduce rice embryo viability (Figure 7A), indicating that coumarin may not inhibit the germination of rice seeds and that the inhibition of ROS accumulation cannot be achieved by reducing the viability of the rice seed embryo. SOD, catalase, and APX are ROS-degrading enzymes and are involved in the regulation of ROS accumulation in plants (Ye et al., 2014). Coumarin increased the SOD and catalase activities, especially at the later stage of seed imbibition, but had no substantial effect on APX activity (Figures 8A,B), indicating that coumarin may specifically enhance the expression of SOD and catalase, thereby accelerating ROS degradation in the embryo. However, the mechanism remains to be further studied.

There is crosstalk between ROS and phytohormones in seed germination. ROS production is increased by ABA in stressed plants (Gechev et al., 2006), but the regulatory role of ABA on ROS production differs between seed and non-seed tissues (Ye et al., 2012). During seed germination of cress and rice, ABA causes a decrease in •OH radical production in seeds (Müller et al., 2009). In *Hedysarum scoparium* seeds, ABA treatment significantly inhibited germination and reduced the H_2_O_2_ content in both cold-stratified and non-cold-stratified seeds (Su et al., 2016). In our present study, coumarin, which inhibits germination, increased the ABA content while decreasing the ROS content of the embryo. In conclusion, our results enhance our understanding of the regulation of ABA and ROS levels during seed germination, although the underlying mechanisms need further study. Furthermore, our research will probably contribute to farming, such as the use of coumarin as a practical compound to prevent viviparous germination of crops.

## Data Availability

All datasets generated for this study are included in the manuscript and/or the Supplementary Files.

## Author Contributions

JL, B-XC, and HF designed the research. B-XC, Y-XP, J-DG, QZ, and Q-JL performed the experiments. B-XC analyzed the data and wrote the manuscript. JL and B-XC critically revised the manuscript.

### Conflict of Interest Statement

The authors declare that the research was conducted in the absence of any commercial or financial relationships that could be construed as a potential conflict of interest.

## Footnotes

^1^https://itol.embl.de/

^2^http://smart.embl-heidelberg.de/

## Supplementary Material

SUPPLEMENTARY FIGURE S1 Effect of coumarin, ABA biosynthesis inhibitor, and (or) ABA catabolic inhibitor on the germination percentage of rice seeds. Data represent means ± SE of three biological replicates of 100 seeds each. Means denoted by the same letter did not significantly differ at *p* < 0.05 according to Fisher’s least significant difference test.

SUPPLEMENTARY FIGURE S2 Expression profiles for rice OsNCED1–5 and OsABA8’ox2/3 genes. Expression profiles, shown as heat maps, for rice OsNCED1–5 and OsABA8’ox2/3 genes during the different periods of rice growth **(A)** and seed germination **(B)**. Expression profiles were obtained from rice microarray data (Os_51k array) as reported in Genevestigator V3. Expression profiles for OsABA8’ox1 were unavailable. The white/blue **(A)** and green/red **(B)** colors reflect the relative expression levels, with dark green representing strong downregulation and dark red representing strong upregulation.

SUPPLEMENTARY TABLE S1 ABA metabolism-related genes in *Oryza sativa, Arabidopsis thaliana,* and other plant species.

SUPPLEMENTARY TABLE S2 Primer sequences for qPCR of *OsGAPDH1, OsNCEDs*, and *OsABA8’oxs.*
