# Supplementary figures and images for: Coumarin-Induced Delay of Rice Seed Germination Is Mediated by Suppression of Abscisic Acid Catabolism and Reactive Oxygen Species Production
Source: Front Plant Sci. 2019 Jun 27;10:828. doi: 10.3389/fpls.2019.00828 (PMC6609317; doi:10.3389/fpls.2019.00828)

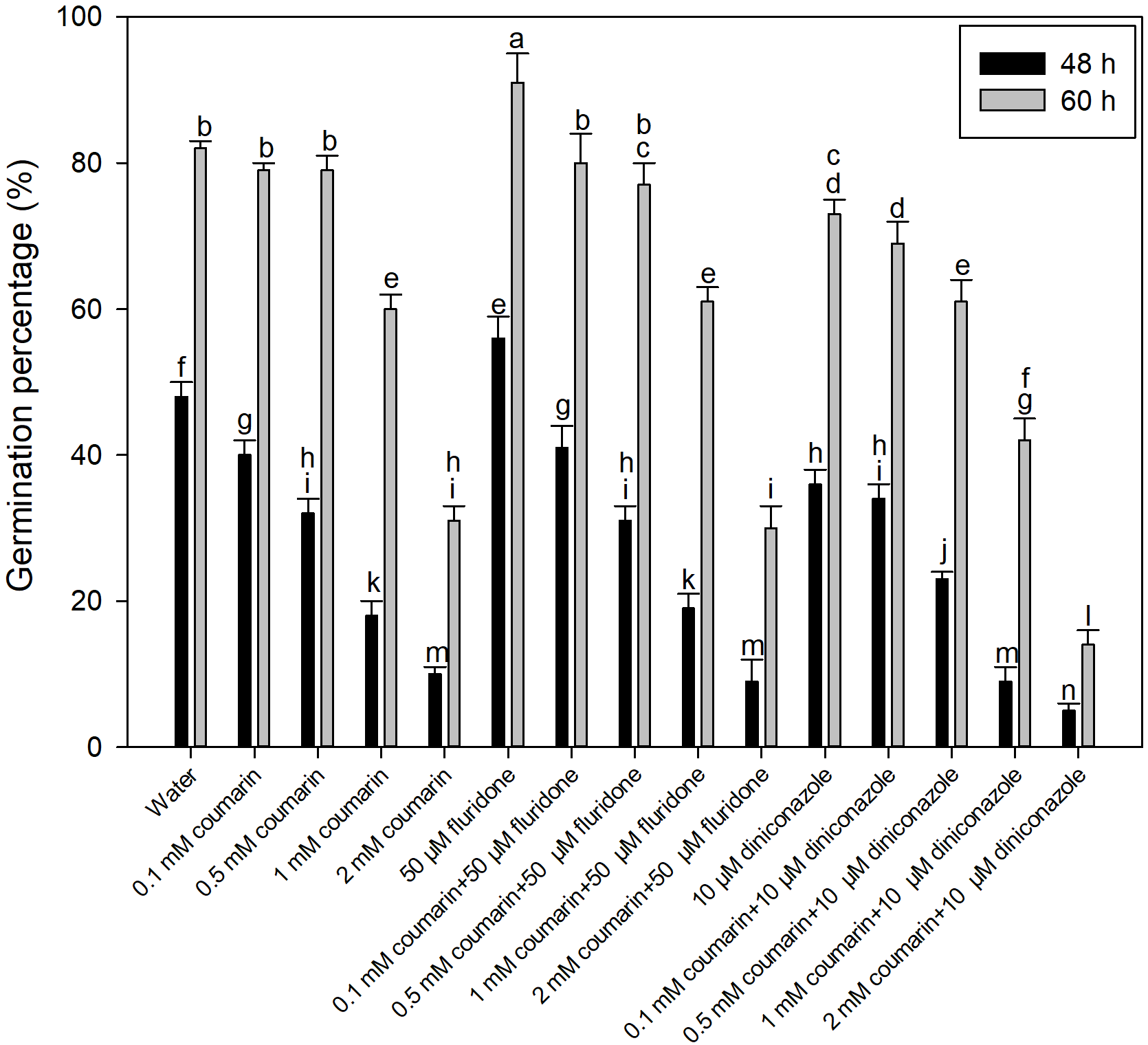

Supplement: SUPPLEMENTARY FIGURE S1 — Effect of coumarin, ABA biosynthesis inhibitor, and (or) ABA catabolic inhibitor on the germination percentage of rice seeds. Data represent means ± SE of three biological replicates of 100 seeds each. Means denoted by the same letter did not significantly differ at p < 0.05 according to Fisher’s least significant difference test. [file Image_1.tif]

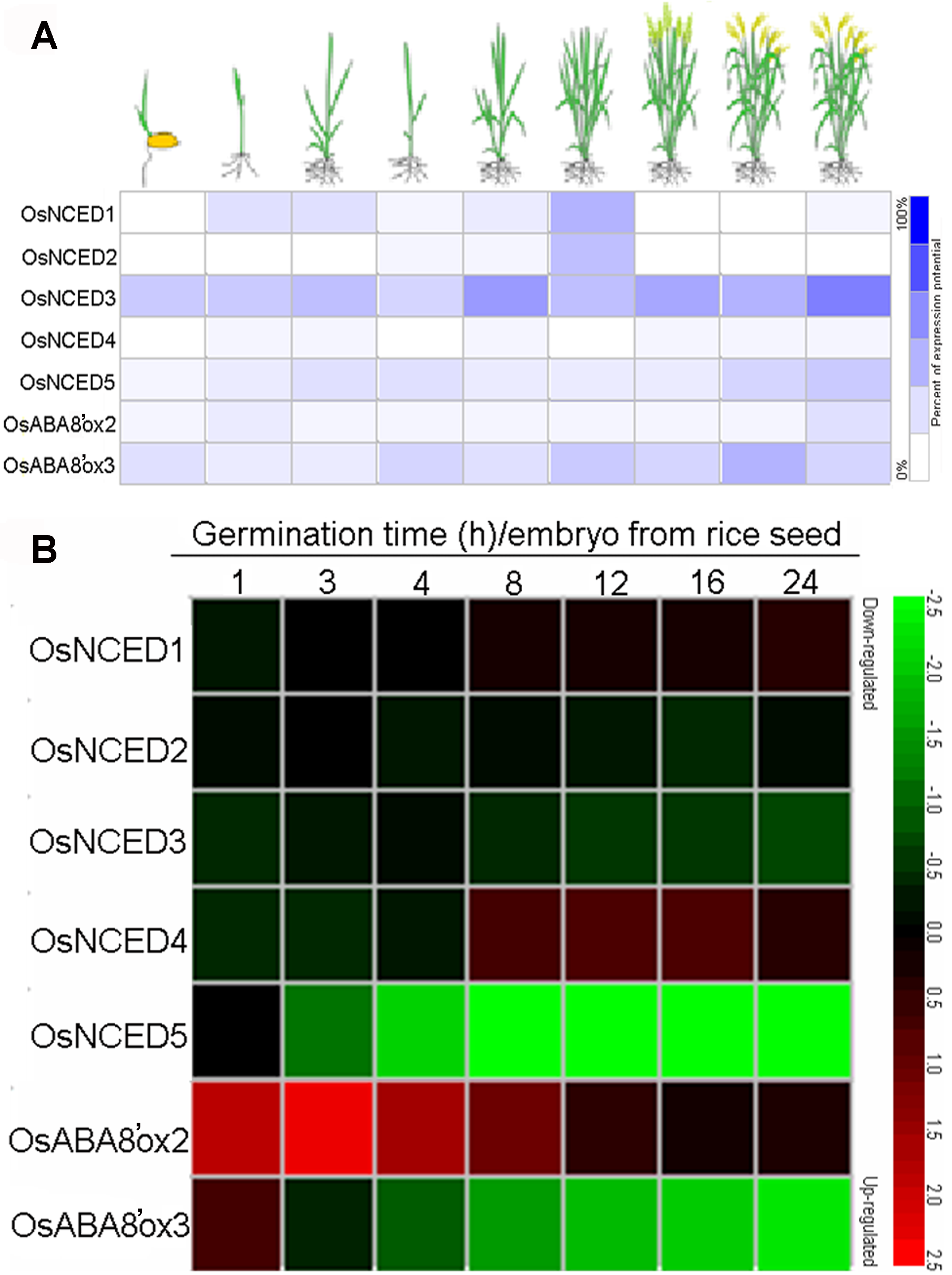

Supplement: SUPPLEMENTARY FIGURE S2 — Expression profiles for rice OsNCED1–5 and OsABA8’ox2/3 genes. Expression profiles, shown as heat maps, for rice OsNCED1–5 and OsABA8’ox2/3 genes during the different periods of rice growth (A) and seed germination (B). Expression profiles were obtained from rice microarray data (Os_51k array) as reported in Genevestigator V3. Expression profiles for OsABA8’ox1 were unavailable. The white/blue (A) and green/red (B) colors reflect the relative expression levels, with dark green representing strong downregulation and dark red representing strong upregulation. [file Image_2.tif]
