# Supplementary material for: Coumarin-Induced Delay of Rice Seed Germination Is Mediated by Suppression of Abscisic Acid Catabolism and Reactive Oxygen Species Production
Source: Front Plant Sci. 2019 Jun 27;10:828. doi: 10.3389/fpls.2019.00828 (PMC6609317; doi:10.3389/fpls.2019.00828)
Supplement: SUPPLEMENTARY TABLE S1 — ABA metabolism-related genes in Oryza sativa, Arabidopsis thaliana, and other plant species. [file Data_Sheet_1.pdf]

Number	Gene number	Species	NCBI gene accession number	NCBI protein Accession number	CDS sequence length (bp)	Domain	Reference
**ABA BIOSYNTHESIS**							
1	*OsNCED1*	*Oryza sativa* L. ssp. *japonica*	AY838897	XP_015626662	1917	RPE65	
2	*OsNCED2*	*Oryza sativa* L. ssp. *japonica*	AY838898	XP_015619611.1	1731	RPE65	
3	*OsNCED3*	*Oryza sativa* L. ssp. *japonica*	AY838899	XP_015631538.1	1872	RPE65	Liu et al., 2011
4	*OsNCED4*	*Oryza sativa* L. ssp. *japonica*	AY838900	XP_015645858.1	1749	RPE65	
5	*OsNCED5*	*Oryza sativa* L. ssp. *japonica*	AY838901	XP_015618707.1	1842	RPE65	
6	*AtNCED2*	*Arabidopsis thaliana*	NM_117945	NP_193569.1	1751	RPE65	
7	*AtNCED3*	*Arabidopsis thaliana*	NM_112304	NP_188062.1	1800	RPE65	Tan et al., 2003;
8	*AtNCED5*	*Arabidopsis thaliana*	NM_102749	NP_174302.1	1770	RPE65	Liu et al., 2011
9	*AtNCED6*	*Arabidopsis thaliana*	NP_189064.1	NP_189064.1	1734	RPE65	
10	*AtNCED9*	*Arabidopsis thaliana*	NM_106486	NP_177960.1	1974	RPE65	
11	*ZmNCED1*	*Zea mays*	NM_001112432	NP_001105902	1815	RPE65	
12	*ZmNCED2*	*Zea mays*		ONM02149	1554	RPE65	
13	*ZmNCED3*	*Zea mays*	NM_001154055	NP_001147527	1896	RPE65	
14	*ZmNCED4*	*Zea mays*	XM_008672757	XP_008670979	1728	RPE65	Voisin et al., 2006
15	*ZmNCED5*	*Zea mays*	NM_001154309	NP_001147781	1722	RPE65	Alexandrov et al., 2009
16	*ZmNCED6*	*Zea mays*	NM_001196164	NP_001183093	1917	RPE65	Schnable et al., 2009
17	*ZmNCED7*	*Zea mays*		ONM61280		RPE65	
18	*ZmNCED8*	*Zea mays*	XM_008647905	XP_008646127	2097	RPE65	
19	*ZmNCED9*	*Zea mays*	XM_008646423	XP_008644645.1	1806	RPE65	
20	*CmNCED1*	*Cucumis melo*	EU180589	ABW80854	740 (partial cds)	RPE65	Sun et al., 2013
21	*CmNCED4a*	*Chrysanthemum x morifolium*	AB247159	BAF36655	1806	RPE65	Ohmiya et al., 2006
22	*CmNCED4b*	*Chrysanthemum x morifolium*	AB247161	BAF36657	1749	RPE65	
23	*PpNCED1*	*Prunus persica*	EF625684	ABV01922	740 (partial cds)	RPE5	Zhang et al., 2009
24	*PpNCED2*	*Prunus persica*	EU912386	ACL00682	742 (partial cds)	RPE5	
25	*DkNCED1*	*Diospyros kaki*	EU925812	ACL00684	740 (partial cds)		Leng et al., 2009
26	*LeNCED1*	*Solanum lycopersicum*	NM_001247526	NP_001234455	1818	RPE65	Nitsch et al., 2009

**ABA CATABOLISM**							
27	*OsABA8'ox1 (OsCYP707A5)*	*Oryza sativa* L. ssp. *japonica*	AB277270	BAF34848	1416	P450	Liu et al., 2011<br>Saika et al., 2007
28	*OsABA8'ox2 (OsCYP707A6)*	*Oryza sativa* L. ssp. *japonica*	NM_001068556	XP_015648451	1521	P450	
29	*OsABA8'ox3* (OsCYP707A7)	*Oryza sativa* L. ssp. *japonica*	NM_001069901	XP_015610835	1503	P450	
30	*AtCYP707A1*	*Arabidopsis thaliana*	AT4G19230	NP_567581	1404	P450	Umezawa et al., 2006<br>Millar et al., 2006
31	*AtCYP707A2*	*Arabidopsis thaliana*	AT2G29090	XP_020885163	1458	P450	
32	*AtCYP707A3*	*Arabidopsis thaliana*	AT5G45340	OAO93915	1722	P450	
33	*AtCYP707A4*	*Arabidopsis thaliana*	AT3G19270	NP_001319589	1332	P450	
34	*PtCYP707A4*	*Populus trichocarpa*	XM_002313817	XP_002313853	1424	P450	Zheng et al., 2012
35	*LsABA8ox1*	*Lactuca sativa*	AB235917	BAG12741	1395	P450	Sawada et al., 2008
36	*LsABA8ox2*	*Lactuca sativa*	XM_023904641	XP_023760409	1455	P450	
37	*LsABA8ox3*	*Lactuca sativa*	AB235919	BAG12743	1404	P450	
38	*LsABA8ox4*	*Lactuca sativa*	AB235920	BAG12744	1482	P450	
39	*GmCYP707A1a*	*Glycine max*	EF377341	ABQ65856	1407	P450	Zheng et al., 2012
40	*GmCYP707A1b*	*Glycine max*	XM_003534293	XP_003534341	1407	P450	
41	*NtCYP707A*	*Nicotiana tabacum*	NM_001325315	NP_001312244	1437	P450	Simon-Mateo et al., 2006
42	*StCYP707A1*	*Solanum tuberosum*	NM_001288216	NP_001275145	1410	P450	Suttle et al., 2013
43	*PvCYP707A1*	*Phaseolus vulgaris*	DQ352541	ABC86558	1395	P450	Yang and Zeevaart, 2006
44	*PvCYP707A2*	*Phaseolus vulgaris*	DQ352542	ABC86559	1398	P450	
45	*PvCYP707A3*	*Phaseolus vulgaris*	DQ352543	ABC86560	1458	P450	
46	*Zm00001d051554*	*Zea mays*	NM_001346919	NP_001333848	1428	P450	Schnable et al., 2009
47	*Zm00001d050021*	*Zea mays*	NM_001347215	NP_001334144	1515	P450	
48	*Zm00001d005889*	*Zea mays*		ONM22327		P450	
49	*Zm00001d017762*	*Zea mays*	NM_001176331	NP_001169802	1413	P450	
50	*Zm00001d020717*	*Zea mays*	NM_001137126	NP_001130598	1500	P450	

REFERENCES

Alexandrov, N., Brover, V., Freidin, S., Troukhan, M., Tatarinova, T., Zhang, H, et al. (2009). Insights into corn genes derived from large-scale cDNA sequencing. *Plant Mol. Biol.* 69 (1-2), 179-194. doi: 10.1007/s11103-008-9415-4

Leng, P. , Zhang, G. L. , Li, X. X. , Wang, L. H. , and Zheng, Z. M. (2009). Cloning of 9-cis-epoxycarotenoid dioxygenase (nced) gene encoding a key enzyme during abscisic acid (aba) biosynthesis and aba-regulated ethylene production in detached young persimmon calyx. *Chin. Sci. Bull.* 54(16), 2830-2838. doi.org/10.1007/s11434-009-0685-2

Liu, F., Zhang, H., Wu, G., Sun, J., Hao, L., Ge, X., et al. (2011). Sequence variation and expression analysis of seed dormancy-and germination-associated ABA- and GA-related genes in rice cultivars. *Front Plant Sci.* 2:1-13. doi.org/10.3389/fpls.2011.00017

Nitsch, L., Oplaat, C., Feron, R., Ma, Q., Wolters-Arts, M., Hedden, P., et al. (2009). Abscisic acid levels in tomato ovaries are regulated by *LeNCED1* and *SlCYP707A1*. *Planta* 229, 1335–1346. doi.org/10.1007/s00425-009-0913-7

Millar, A., Jacobsen, J., Ross, J., Helliwell, C., Poole, A., Scofield, G., et al. (2006). Seed dormancy and ABA metabolism in Arabidopsis and barley: the role of ABA 8’-hydroxylase. *Plant J.* 45:9542-954. doi: 10.1111/j.1365-313X.2006.02659.x

Tan, B-C., Joseph, L., Deng, W-T., Liu, L., Li, Q-B., Cline, K., et al. (2003). Molecular characterization of the *Arabidopsis 9-cis* epoxycarotenoid dioxygenase gene family. *Plant J.* 35:44-56. doi: 10.1046/j.1365-313X.2003.01786.x

Umezawa T, Okamoto M, Kushiro T, Nambara E, Oono Y, Seki M., et al. (2006). CYP707A3, a major ABA 8’-hydroxylase involved in dehydration and rehydration response in *Arabidopsis thaliana*. *Plant J*. 46:171-182. doi: 10.1111/j.1365-313X.2006.02683.x

Ohmiya, A., Kishimoto, S., Aida, R., Yoshioka, S., and Sumitomo, K. (2006). Carotenoid cleavage dioxygenase (CmCCD4a) contributes to white color formation in chrysanthemum petals. *Plant Physiol.* 142:1193-1201. doi/10.1104/pp.106.087130

Saika, H., Okamoto, M., Miyoshi, K., Kushiro, T., Shinoda, S., Jikumaru, Y., et al. (2007). Ethylene promotes submergence-induced expression of *OsABA8ox1*, a gene that encodes ABA 8’-hydroxylase in rice. *Plant Cell Physiol*. 48 (2):287-298. doi:10.1093/pcp/pcm003

Sawada, Y., Aoki, M., Nakaminami, K., Mitsuhashi, W., Tatematsu, K., Kushiro, T., et al. (2008). Phytochrome- and gibberellin-mediated regulation of abscisic acid metabolism during germination of photoblastic lettuce seeds. *Plant Physiol.* 146: 1386-1396. doi: 10.1104/pp.107.115162

Schnable, P., Ware, D., Fulton, R., Stein, J., Wei, F. et al. (2009). Pasternak SThe B73 maize genome: complexity, diversity, and dynamics. *Science*. 337(6098):1040. doi: 10.1126/science.1178534

Simon-Mateo, C., Depuydt, S., Oliveira Manes, C., Cnudde, F., Holsters, M., Goethals, K., et al. (2006). The phytopathogen Rhodococcus fascians breaks apical dominance and activates axillary meristems by inducing plant genes involved in hormone metabolism. *Mol. Plant Pathol.* 7 (2), 103-112.

doi.org/10.1111/j
Sun, Y., Chen, P., Duan, C., Tao, T., Wang, Y., Ji, K., et al (2013). Transcriptional regulation of genes encoding key enzymes of abscisic acid metabolism during melon (*Cucumis melo* L.) fruit development and ripening. *J. Plant Growth Regul*. 32: 233. doi.org/10.1007/s00344-012-9293-5
Suttle, J., Lulai, E., Huckle, L. and Neubauer, J. (2013). Wounding of potato tubers induces increases in ABA biosynthesis and catabolism and alters expression of ABA metabolic genes. *J Plant Physiol*. 170: 560-566. doi: org/10.1016/j.jplph.2012.11.012
Voisin, A., Reidy, B., Parent, B., Rolland, G., Redondo, E., Gerentes, D., et al. (2006). Are ABA, ethylene or their interaction involved in the response of leaf growth to soil water deficit? An analysis using naturally occurring variation or genetic transformation of ABA production in maize. *Plant Cell Environ.* 29 (9), 1829-1840. doi: org/10.1111/j
Yang, S. and Zeevaart, J. (2006). Expression of ABA 8'-hydroxylases in relation to leaf water relations and seed development in bean. *Plant J*. 47: 675-686. doi: 10.1111/j.1365-313X.2006.02815.x
Zhang, M., Leng, P., Zhang, G. and Li, X. (2009). Cloning and functional analysis of 9-cis-epoxycarotenoid dioxygenase (NCED) genes encoding a key enzyme during abscisic acid biosynthesis from peach and grape fruits. *J. Plant Physiol.* 166 (12):1241-1252. doi.org/10.1016/j.jplph.2009.01.013
Zheng, Y., Huang, Y., Xian, W., Wang, J. and Liao. H. (2012). Identification and expression analysis of the *Glycine max CYP707A* gene family in response to drought and salt stress. *Ann. Bot.* 110: 743 –756. doi:10.1093/aob/mcs133
