# Supplementary material for: Coumarin-Induced Delay of Rice Seed Germination Is Mediated by Suppression of Abscisic Acid Catabolism and Reactive Oxygen Species Production
Source: Front Plant Sci. 2019 Jun 27;10:828. doi: 10.3389/fpls.2019.00828 (PMC6609317; doi:10.3389/fpls.2019.00828)
Supplement: SUPPLEMENTARY TABLE S2 — Primer sequences for qPCR of OsGAPDH1, OsNCEDs, and OsABA8’oxs. [file Data_Sheet_2.pdf]

SUPPLEMENTARY TABLE 2 | Primer sequences for qPCR of *OsGAPDH1*, *OsNCEDs*, and *OsABA8'oxs*

Gene	Locus	Primer sequence	Product length
OsNCED1	LOC_Os02g47510	Forward: 5'-GAACTTCGACTTCCCCGTGA-3'<br>Reverse: 5'- ACGTAGCACACCAAGTACCC-3'	252
OsNCED2	LOC_Os12g24800	Forward: 5'-GAGCCTTGAGTTCGGTGTCA-3'<br>Reverse: 5'- CAGCAAAGCACCCTAGACCA-3'	179
OsNCED3	LOC_Os03g44380	Forward: 5'-ATATGGCGACGATCACGACG-3'<br>Reverse: 5'-CGCGGAGAATCTCACCGAAT-3'	110
OsNCED4	LOC_Os07g05940	Forward: 5'-TCGGGAGGTACGACTTCCAT-3'<br>Reverse: 5'-TTGAGGTACGGCTTGGACAC-3'	127
OsNCED5	LOC_Os12g42280	Forward: 5'-CGAGCTCACCAAGTTCGAGT-3'<br>Forward: 5'-TTGATGAAGGTGCCGTGGAA-3'	249
OsABA8'ox1	LOC_Os02g47470	Forward: 5'-AAAACCAACATCAACGGCGG-3'<br>Forward: 5'-GATTGCCAACCGTGGTCCTA-3'	189
OsABA8'ox2	LOC_Os08g36860	Forward: 5'-CGTGTGTGTGGATGCAATGG-3'<br>Forward: 5'-AGTGCTACACTAGGCACACC-3'	213
OsABA8'ox3	LOC_Os09g28390	Forward: 5'-CTGGTCACTGGCTACAGGTG-3'<br>Forward: 5'-TGCTACGCCATTGTCGTCAT-3'	168
OsGAPDH1	LOC_Os02g38920	Forward: 5'-GCAATCAAGGAGGAGGCTGA-3'<br>Reverse: 5'-ACGTGTCGCTCAAAGCAATG-3'	139
